# Influence of sex on the exposure to isoniazid in patients with pulmonary tuberculosis

**DOI:** 10.1590/S1678-9946202365056

**Published:** 2023-10-20

**Authors:** Juan Gonzalo Bardalez Rivera, Carlos Augusto Abreu Albério, José Luiz Fernandes Vieira

**Affiliations:** 1 Universidade Federal do Pará, Faculdade de Farmácia, Belém, Pará, Brazil; 2 Hospital Universitário João de Barros Barreto, Ambulatório de Tuberculose Multiressistente, Belém, Pará, Brazil

**Keywords:** Tuberculosis, Isoniazid, Infectious diseases, Drug monitoring, Antituberculosis drugs

## Abstract

Isoniazid is a key component of tuberculosis treatment. Adequate exposure is a determinant for therapeutic success; however, considerable inter- and intraindividual variations in drug plasma levels can lead to unfavorable outcomes. While some predictors of isoniazid levels are well-known, others, such as sex, yield controversial results, requiring further investigation to optimize exposure. This study investigates whether the sex of patients influences the dose administered and the concentrations of isoniazid in plasma. Levels of isoniazid were associated with the N-acetyltransferase 2 phenotypes. A total of 76 male and 58 female patients were included. Isoniazid was measured by high-performance liquid chromatography, and N-acetyltransferase 2 phenotypes were assessed using molecular techniques. The results show that the dose administered, expressed in mg/kg, was higher in females, but the plasma levels were similar between both sexes. Among patients, 46.2%, 38.8%, and 15% were slow, intermediate, and fast acetylators, respectively. As expected, isoniazid levels were associated with the acetylation phenotypes, with higher concentrations in the slow acetylators. Thus, sex-related difference in isoniazid levels is due to the body weight of patients, and the optimized dose regimen based on patient weight and acetylator phenotypes can improve the treatment outcomes.

## INTRODUCTION

Tuberculosis is still an important public health problem in Brazil, with 68,271 cases reported in 2021. In the Para State, the coefficient of incidence was 42.6 per 100,000 inhabitants, which is higher than the national average of 32.0 per 100,000 inhabitants. Mortality rate was also high in Para, with a coefficient of 2.8 per 100,000 inhabitants^1^. Isoniazid (INH) is a key component of the first-line treatment for pulmonary tuberculosis^2^. Achieving adequate drug exposure is a decisive factor for therapeutic success^3,4^. However, the pharmacokinetics of INH exhibit significant intra- and interindividual variations, which can affect its plasma concentrations. This may result in insufficient drug exposure to the bacilli, leading to increased rates of unsatisfactory outcomes^3-5^.

The plasma concentration of INH is influenced by several variables, which encompass severity of tuberculosis, comorbidities like HIV coinfection, concurrent food intake, N-acetyltransferase 2 (NAT2) acetylation phenotypes, and the quality of the dispensed medication. A comprehensive understanding and effective management of these variables are indispensable for achieving favorable treatment outcomes, preventing relapses, reducing drug resistance, and ultimately alleviating the burden of the disease^5-8^.

The influence of sex on antituberculosis plasma levels is a subject of significant interest since it can affect treatment outcomes and rates of adverse effects. Certain drugs may show substantial alterations in their plasma levels between sexes due to variations in the hormonal cycle, the use of oral contraceptives, pregnancy, body weight, and fat distribution^9^. Moreover, sex-related differences can also impact the appropriate drug dosage^9,10^. Regarding INH, there are conflicting data, as some studies have reported greater exposure in female patients^5-7^, while others have observed a similar pattern of drug exposure between sexes^8-10^.

In Brazil, there are few reports on the administered dose of antituberculosis drugs based on patient body weight and their plasma concentrations. Given that the therapeutic efficacy relies on INH exposure^4,5^, evaluating its drug plasma levels and the potential influence of sex contributes to optimizing treatment^10^. The primary aim of the study is to investigate whether the sex of patients affects the administered dose and plasma concentrations of INH. The association between plasma levels of INH and acetylator phenotypes, and their relationship with the percentage of patients who experience sputum conversion during the intensive phase of treatment also were assessed.

An observational study was conducted from January to December 2018 in the Basic Health Unit of the Guama District in the Belem municipality, Brazil. Adult patients of both sexes with clinical, laboratory, and radiological diagnostic of pulmonary tuberculosis under ambulatory treatment were eligible for inclusion. The following exclusion criteria were used: positive laboratory tests for HIV, hepatitis A, B, C, or E; drug addiction including alcohol and tobacco; diabetes mellitus; or previous reports of allergy to antituberculosis drugs. The treatment follows the recommendations of the World Health Organization (WHO): a daily total dose of rifampicin (600 mg), isoniazid (300 mg), pyrazinamide (1,600 mg), and ethambutol (1,100 mg) for two months (intensive phase); and rifampicin (600 mg) with isoniazid (300 mg) for four months (continuation phase)^2^. Drugs were dispensed as a fixed-dose combination in both phases. The research team supervised the first monthly dose. Then, a family member supervised the daily administration of drugs. The quality of the drugs used in the study was ensured by Farmanguinhos-Fiocruz/RJ, and the drugs were from the same manufacturer and batch.

### Ethical statement

The Ethical Committee of the Health Science Institute of the Universidade Federal do Para revised and approved the study under the Nº 1.591.019. All patients provided informed consent before inclusion in the study.

## MATERIALS AND METHODS

Blood samples were collected from each patient in heparinized tubes on day 60 of follow-up after a 12-h fast and 2 h post-dosing. A portion of the sample was centrifuged at 3,500rpm×10min at 4 °C for plasma separation and frozen at -20 °C for drug analysis. The remaining blood was used for genotyping of N-acetyltransferase 2 (NAT-2).

Isoniazid was measured by reverse-phase high-performance liquid chromatography using a Flexar^®^ system (Perkin Elmer), after liquid-liquid extraction from plasma, following the procedure proposed by Prasanthi et al.^11^. Limits of detection and quantification were 0.01 μg/mL and 0.02 μg/mL, respectively. The method was linear from 0.02 to 20 μg/mL. The within and between days coefficients of variation were 12 and 16%, respectively, and the mean recovery was 90%.

Study participants were classified into three NAT2 acetylator phenotypes: slow, intermediate, and fast, based on their genotypes. Fast acetylation was considered if the genotype contained two fast alleles (NAT24, NAT213, NAT211, and NAT212). Slow acetylation was considered if the genotype contained two slow alleles (NAT25, NAT26, NAT27, and NAT214). Lastly, intermediate acetylator phenotype for genotype containing one slow and one fast acetylator allele. To infer the human NAT2 acetylator phenotype, the NAT2PRED software program was used^12,13^. The frequency of each *NAT2* acetylator phenotype was also calculated.

Extraction of genomic DNA was performed with the Wizard^®^ Genomic DNA purification kit (Promega, Madison, USA) using standard procedure. The *NAT2* gene polymorphisms were studied by 986 base-pair (bp) sequencing amplified in two independent polymerase chain reactions (PCR), with the following primers: NAT2-1F (5’-TTA ATT CTC ATC TCC TGC CAA AGA-3’), NAT2-1R (5’-TCA CTC TGC TTC CCA AGA TAA TCA-3’); NAT2-2F (5’-ATG GAG TTG GGC TTA GAG GCT AT-3’), NAT2-2R (5’-CTT TGG CAG GAG ATG AGA ATT AAG A-3’). Amplification was performed using a standard protocol. PCR products were purified using the Purelink kit (Invitrogen Life Technologies, Carlsbad, CA, USA). Sequencing was conducted on an ABI PRISM 3130 Genetic Analyzer (Applied Biosystems, Foster City, CA, USA). Sequence analyses were performed using Codon Code Aligner, version 3.0.1 (Codon Code Corporation, Centerville, MA, USA).

## DISCUSSION

The study assumed that a 20% difference in the plasma concentration of INH between sexes could alter the exposure to the drug, considering a mean concentration of 3.25 µg/mL isoniazid, a 0.7 µg/mL standard deviation, and the desired plasma concentrations of 3 to 5 µg/mL for sensitive strains of *Mycobacterium tuberculosis* in both sexes. It was estimated that a minimum of 18 patients would provide at least 80% power to detect a 20% difference in the plasma concentration of INH between male and female patients, with 95% confidence^3-5^.

Data are presented as frequencies of occurrence or as mean (95% confidence interval). Chi-square (X^2^) was used to compare the qualitative variables between sexes. The Student’s *t*-test was used to compare the variables between sexes. A bivariate analysis was performed to associate the concentrations of INH with the *NAT2* acetylator phenotypes and the sex of the patients. Allele and genotype frequencies were estimated by gene counting. Deviation from Hardy-Weinberg equilibrium was assessed by the Chi-square test with Bonferroni correction using the Genepop software package (version 4.7.0, Genepop C++). A 5% significance level was accepted.

A total of 134 patients were included in this study. Male patients accounted for 76 (56.7%) of the total. Mean ages were 42 (25–59) years and 42 (21–53) years for males and females (t= 3.75; p=0.842), respectively. The mean weights were 72 (63–78) kg in males and 60 (50–67) kg in females (t= 8.93; p<0.001).

Mean daily doses of INH administered based on patient weight were 4.2 (3.8–4.7) mg/kg and 4.8 (4.6–5.1) mg/kg in males and females, respectively (t=16.2; p<0.0001). Females received approximately 12.5% more INH than male patients. This difference can be attributed to the significant difference in body weight between sexes, with lower values observed in female patients. Supporting this finding, a previous study demonstrated that females could potentially receive up to 19.1% more INH than males^14^. Moreover, it is essential to highlight that previous studies have reported higher rates of adverse effects resulting from antituberculosis drugs in females^10,15^.

Mean daily doses of INH received by both male and female patients were within the range recommended by WHO, which suggests a dose of 5 (4–6) mg/kg of body weight^2^. During the study period, patients weighing over 50 kg received a daily dose of 300 mg, following the guidelines of the Brazilian National Program for Control of Tuberculosis. Recognizing the significance of body weight in enhancing treatment outcomes, the Brazilian Program adjusted weight bands in 2019. Accordingly, patients with body weights ranging from 51 to 70 kg were prescribed a daily dose of 300 mg, and those weighing over 70 kg were given 450 mg. However, it is noteworthy that these weight bands might require further expansion above 70 kg due to the prevalent issues of overweight and obesity in Brazil^16^.

Genotyping revealed that 46.2%, 38.8%, and 15% of both males and females were slow, intermediate, and fast acetylators (X^2^=15.94; p=0.0003), respectively. Genotype distribution did not deviate significantly from the Hardy-Weinberg equilibrium (p>0.05).

The frequency of acetylator phenotypes among study patients aligns with another investigation conducted in a similar population, which reported a 56.7% prevalence for the slow acetylator phenotype. In White and Black/Mixed-race populations, most individuals carry at least one copy of a slow acetylator allele, and less than 10% are homozygous for the fast acetylator phenotype^12^.

The pharmacokinetic parameter used for estimating the exposure to INH was the maximum concentration (C_max_), which is typically achieved around 2h after dosing in fasting patients^3-6^. However, there is an interesting discussion regarding the appropriate pharmacokinetic parameter to estimate exposure to antituberculosis drugs. It has been suggested that, for drugs characterized by a short half-life and variable oral bioavailability, a limited sampling strategy involving two or three time points to estimate the area under the concentration-time curve (AUC) is the most effective approach for assessing drug exposure. This strategy also enables differentiation between delayed absorption and malabsorption^7,17^.

However, conducting multiple blood sampling in the ambulatory setting poses various logistical and financial challenges. Therefore, the 2-hour post-dose concentration of INH can be a suitable alternative to assess drug exposure. This time point approximates the C_max_ for most antituberculosis drugs and has been linked to favorable treatment outcomes^3-6^.

The C_max_ of INH were found to be comparable between males and females in each phenotype studied, ranging from 1.6 to 4.6 µg/mL. The minor increase in drug levels in female patients can be attributed to the differences in the dose administered between sexes^4,14^. Despite the evidence that men are disadvantaged in seeking and/or accessing tuberculosis care, it is also plausible that the low concentrations of INH and the low dose administered may contribute to poorer treatment outcome in males^18,19^.

As anticipated, the NAT2 polymorphism emerged as a significant predictor of plasma drug levels in both sexes^4,6,14^. Plasma concentrations of 3 to 5 µg/mL are adequate for sensitive strains of *M. tuberculosis*. Fast acetylators presented the lowest levels of INH compared to the intermediate and slow acetylators in both sexes (p<0.001). Table 1 shows the plasma INH concentrations at 2h post-dosing in male and female patients and the respective acetylator phenotypes.

**Table 1:** Plasma concentrations of isoniazid in patients with pulmonary tuberculosis according to sex and N-acetyltransferase phenotype.

Sex	n	Isoniazid (µg/mL) Fast	n	Isoniazid (µg/mL) Intermediate	n	Isoniazid (µg/mL) Slow
Male	12	1.87 (1.7–2.1)	29	3.5 (2.1–3.9)	35	3.97 (3.7–4.1)
Female	12	2.01 (1.6–3.3)	20	3.9 (2.5–4.3)	26	4.24 (3.8–4.6)

Males and females with the slow acetylator phenotype have INH plasma levels within the desired concentrations^3,4^, whereas 62.5% and 66.7% of males and females with intermediate acetylator phenotype presented drug levels within the adequate range. Only 33% of females with the fast acetylator phenotype have drug plasma levels within the expected concentrations, whereas all males have values below the optimal range. This finding is consistent with previous studies that have reported similar high percentages, with up to 68% of patients having these phenotypes showing suboptimal INH levels after receiving daily doses of 300 and 450 mg. These results indicate underexposure to INH and emphasize the need for adjusting the dosage regimen based on acetylator phenotypes^4,6,14,20^.

The bivariate analysis associating the acetylator phenotype and sex with isoniazid levels showed that the concentrations of INH were significantly associated with the *NAT2* acetylator phenotypes (F=19.58; p<0.001), but not with sex (F=1.48; p=0.2284). The interaction between sex and *NAT2* acetylator phenotypes was also not significant (F=0.027; p=0.9728).

Moreover, the standardization of the drug plasma concentrations by the body weight of each patient showed similar results, with a significant association between INH plasma concentrations and *NAT2* acetylator phenotypes (F=16.58; p<0.001), and no significant association with sex (F=0.1534; p=0.6967).

All patients presented sputum conversion after treatment, but there was a significant influence of acetylator phenotypes on the time for sputum conversion. Specifically, 93%, 87%, and 49% of sputum conversion occurred in patients with slow, intermediate, and fast acetylator phenotypes, respectively, at 60 days of treatment (X2=17.31; p=0.0002). Additionally, 15% of individuals with fast acetylators, sputum conversion occurred in the last month of treatment. Based on the drug levels observed in the study, it can be inferred that patients with the fast acetylator phenotype may require approximately twice the dose of INH to achieve plasma concentrations similar to those observed in patients with the slow acetylator phenotype.

Sex-related differences in body weight and acetylator phenotypes can result in suboptimal exposure to INH, contributing to unfavorable treatment outcomes and the emergence of resistance. Increasing the daily dose of INH could be a potential strategy to address this issue. Although concerns have been raised about the potential dose-dependent toxicity of INH, a study provided evidence that patients can tolerate higher doses of the drug, without experiencing significant adverse reactions^20^.

In the clinical setting, when patients receiving an adequate daily dose of isoniazid and demonstrating sensitivity to rifampicin and isoniazid experience a delay in sputum conversion, it raises the possibility of fast or intermediate acetylator phenotypes, and assessment of the *NAT2* phenotype is recommended. A multicenter study conducted in Brazil, India, and South Africa demonstrated that pharmacogenomic-guided therapy is not only cost-effective but also leads to improved health outcomes^20^. However, a limitation arises in the context of the public health system in Brazil, as only molecular tests to detect resistance to isoniazid and rifampicin are available. Consequently, the assessment of acetylator phenotypes may not be feasible in most patients. As a result, the only accessible strategy for clinical physicians to optimize treatment becomes the monthly monitoring of patient weight to adjust the dosage regimen adequately. This approach aligns with the recommendation of WHO, which emphasizes the importance of monthly monitoring patient’s weight during treatment to adjust the dosage regimen adequately^2^.

The main limitation of this study was the small sample of patients with the fast acetylator phenotype, which is a common characteristic of observational studies, in which patients are included based on random selection.

## CONCLUSION

In conclusion, sex-related differences in body weight explain the variations in the dose of INH administered to patients and the slightly higher levels of the drug in females. On the other hand, the *NAT2* phenotype is a significant predictor of isoniazid levels, and a substantial proportion of patients with intermediate and fast acetylator phenotypes were underexposed to the drug, which impacted the time for sputum conversion. Thus, the optimization of the dosage regimen based on patient body weight and acetylator phenotypes can improve the outcomes.
